# Clinical Benefit from Ipilimumab Therapy in Melanoma Patients may be Associated with Serum CTLA4 Levels

**DOI:** 10.3389/fonc.2014.00110

**Published:** 2014-05-16

**Authors:** Anna M. Leung, Agnes Fermin Lee, Junko Ozao-Choy, Romela Irene Ramos, Omid Hamid, Steven J. O’Day, Myung Shin-Sim, Donald L. Morton, Mark B. Faries, Peter A. Sieling, Delphine J. Lee

**Affiliations:** ^1^Melanoma Research Program, John Wayne Cancer Institute at Saint John’s Health Center, Santa Monica, CA, USA; ^2^Dirks/Dougherty Laboratory for Cancer Research, Department of Translational Immunology, John Wayne Cancer Institute at Saint John’s Health Center, Santa Monica, CA, USA; ^3^Melanoma Center, The Angeles Clinic and Research Institute, Los Angeles, CA, USA; ^4^Department of Hematology and Oncology, Beverly Hills Cancer Center, Beverly Hills, CA, USA; ^5^Department of Biostatistics, John Wayne Cancer Institute at Saint John’s Health Center, Santa Monica, CA, USA

**Keywords:** serum, soluble CTLA4, ipilimumab, metastatic melanoma, clinical benefit, survival, response to therapy

## Abstract

Stage IV metastatic melanoma patients historically have a poor prognosis with 5–10% 5-year survival. Ipilimumab, a monoclonal antibody against cytotoxic T-lymphocyte antigen 4 (CTLA4), is one of the first treatments to provide beneficial durable responses in advanced melanoma. However, less than 25% of those treated benefit, treatment is expensive, and side effects can be fatal. Since soluble (s) CTLA4 may mediate inhibitory effects previously ascribed to the membrane-bound isoform (mCTLA4), we hypothesized patients benefiting from ipilimumab have higher serum levels of sCTLA4. We found that higher sCTLA4 levels correlated both with response and improved survival in patients treated with ipilimumab in a small patient cohort [patients with (*n* = 9) and without (*n* = 5) clinical benefit]. sCTLA4 levels were statistically higher in ipilimumab-treated patients with response to ipilimumab. In contrast, sCTLA4 levels did not correlate with survival in patients who did not receive ipilimumab (*n* = 11). These preliminary observations provide a previously unrecognized link between serum sCTLA4 levels and response to ipilimumab as well as to improved survival in ipilimumab-treated melanoma patients and a potential mechanism by which ipilimumab functions.

## Introduction

Cutaneous melanoma is the third most common skin cancer and the most aggressive of all skin cancers with increasing incidence and worsening survival over the past few decades. Only 5–10% of stage IV melanoma patients survive beyond 5 years (1). Ipilimumab is the first FDA approved therapy to show an overall survival (OS) benefit for metastatic melanoma in randomized clinical trials (2).

Ipilimumab is a monoclonal antibody with specificity against cytotoxic T-lymphocyte antigen 4 (CTLA4), an inhibitory immune mediator with both membrane-bound (mCTLA4) and soluble (sCTLA4) forms (3). mCTLA4 is present in intracytoplasmic vesicles of T cells (4) and it is constitutively expressed at low levels in resting T cells (5). CTLA4 is upregulated on activated T cells (6) and translocates to the cell surface to deliver a negative signal to “put the brakes on” T cells (7, 8). CTLA4 is important to maintain self-tolerance and prevent autoimmunity (7, 9, 10). This activity is classically thought to be mediated by the full-length mCTLA4.

In addition to mCTLA4, other alternatively spliced mRNA transcripts of CTLA4 have been detected, including a secretable soluble form, sCTLA4 (3, 11). Recently, sCTLA4 has been shown to be an important immune regulator by inhibiting antigen-specific T cell proliferation and cytokine production (12). Therefore, we hypothesized that patients’ serum levels of sCTLA4 may reflect clinical benefit from treatment with the monoclonal antibody to CTLA4, ipilimumab.

## Materials and Methods

### Identification of patients and patient characteristics

After internal regulatory review approval was obtained, the John Wayne Cancer Institute (JWCI) database was searched for those patients who had received ipilumumab. This database was established in 1971 and currently holds patient clinical characteristics, treatment and outcome data for 14,983 melanoma patients. The database is updated yearly for patient survival and outcome data and in addition is linked with a tissue, peripheral blood cell, and serum biorepository.

We identified 14 patients who received ipilumumab who also had prospectively collected pre-treatment serum available for study. The time range between serum collection and ipilimumab administration was 1–85 months with an average time range of 25 months. Determination of clinical benefit was based on the response evaluation criteria in solid tumors (RECIST), which designates patients having a complete or partial response, stable disease, or progressive disease during treatments. We defined patients with clinical benefit (*n* = 9) as those with complete or partial response or stabilization of disease and patients without clinical benefit (*n* = 5) as those with progression of disease. Scores were determined by serial scans by treating medical and surgical oncologists. Median followup for patients was 8 years.

We also identified Stage IV melanoma patients who had not received ipilimumab (*n* = 11). Serum sCTLA4 levels were measured for this group and correlated with survival. Demographic features from all patients (14 ipilimumab-treated + 11 non-ipilimumab-treated) including age, sex, primary tumor characteristics (anatomical site, Clark level, Breslow depth, ulceration), and additional immune (previous immunotherapy) and surgical treatments (metastectomy) were collected and compared. In addition, complications from ipilimumab treatment were collected from ipilimumab-treated patients.

### Serum CTLA4 measurement

sCTLA4 levels were measured by ELISA (E-bioscience Bender Med System Vienna, Austria), according to the manufacturer’s instructions. Samples were blindly tested in triplicate to generate mean values used in the analysis. Two-tailed Mann–Whitney test was used to determine statistical significance with *p* < 0.05 as statistically significant. The lowest sensitivity threshold was 100 pg/mL. The analytical response was linear approximately between 0.1 and 1.2 of absorbance values corresponding to 100–50,000 pg/mL as assessed by serial dilution test (13). Receiver operating characteristic (ROC) curve analysis was used to determine the cutoff of sCTLA4 levels to classify patients as those with vs. those without clinical benefit. For samples below the limit of detection, a value of 0 pg/mL is arbitrarily assigned.

### Survival analysis

Survival rates were estimated by the Kaplan–Meier method and compared using log-rank test. In addition, both univariable and multivariable Cox proportional hazard regression analyses were performed to examine the association between sCTLA4 level and survival.

### Multivariable analysis

Multivariable analysis was performed to determine the independent association of sCTLA4 levels and survival. Primary outcome measures were ipilimumab benefit, 5-year OS, defined as period from ipilimumab administration until death. Statistical analysis was performed with SAS 9.2 (Cary, NC, USA). *P*-value < 0.05 was considered significant.

## Results

### Characteristics of patients

Characteristics of the patients with benefit from ipilimumab (*n* = 9) and those without benefit from ipilimumab (*n* = 5) are shown in Table 1. Demographics (sex, age, primary location, complications, or M status) and treatments (previous immunotherapy or number of surgeries) were similar between the two groups. Characteristics of the patients who did not receive ipilimumab (*n* = 11) are shown in Table 2.

**Table 1:** **Demographics of ipilimumab-treated melanoma patients with clinical benefit vs. patients without clinical benefit**.

Demographic	Patients with clinical benefit *n* (%)	Patients without clinical benefit *n* (%)	*p*-Value
**Sex**			1.0
Male	5 (55)	3 (60)	
Female	4 (45)	2 (40)	
**Age**			0.51
Average (range)	54 (42–67)	50 (29–66)	
**Previous immunotherapy**			0.41
Yes	6 (67)	4 (80)	
No	3 (33)	1 (20)	
**Complications**			0.50
Yes	2 (22)	0 (0)	
No	7 (78)	5 (100)	
**Metastectomy**			1.00
Yes	6 (67)	4 (80)	
No	3 (33)	1 (20)	
**Primary**			0.33
Trunk	2 (22)	0 (0)	
Mucosal	2 (22)	0 (0)	
Extremity	3 (33)	3 (60)	
Head and neck	2 (22)	1 (20)	
Unknown	0 (0)	1 (20)	
**M status**			0.34
M1a	1 (12)	1 (20)	
M1b	3 (33)	0 (0)	
M1c	5 (55)	4 (80)	
**sCTLA4 level**			0.03
sCTLA4 level <200	2 (22)	4 (80)	
sCTLA4 level >200	7 (78)	1 (20)	

**Table 2:** **Demographics of 11 melanoma patients not treated with ipilimumab**.

Demographic	sCTLA4 ≤200 pg/mL (*n* %)	sCTLA4 >200 pg/mL (*n* %)	*p*-Value
**Sex**			0.34
Male	3 (100)	6 (75)	
Female	0 (0)	2 (25)	
**Age**			0.40
Average	41	52	
**Primary**			0.17
Trunk	2 (66.7)	3 (37.5)	
Extremity	0 (0)	5 (62.5)	
Head and neck	1 (33.3)	0 (0)	
**M status**			0.38
M1a	5 (33.3)	4 (50)	
M1b	0 (0)	2 (25)	
M1c	2 (66.7)	2 (25)	

### Soluble CTLA4 serum levels of melanoma patients

**Figure 1:**
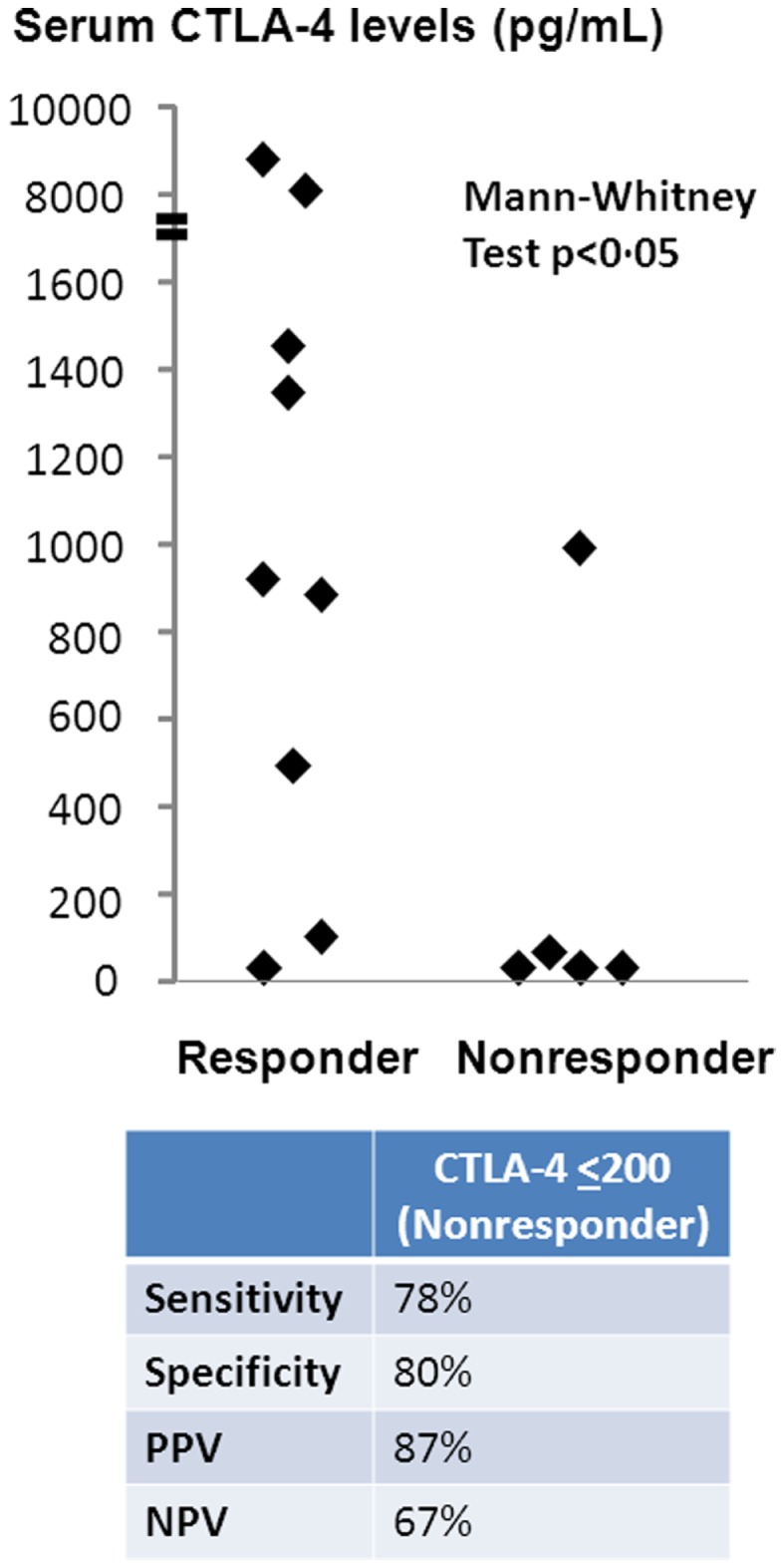
**Serum sCTLA4 levels correlate with clinical benefit to ipilimumab treatment**. sCTLA4 levels were measured by ELISA and individual values plotted according to clinical response. Values are expressed as the mean value of triplicate wells. The sensitivity of the ELISA was 100 pg/mL. Two-tailed Mann–Whitney test was used to evaluate the significance of the differences between patients who received (*n* = 9) or did not receive (*n* = 5) benefit from ipilimumab treatment. The cutoff sCTLA4 level for prediction of clinical benefit was determined using a threshold of 200 pg/mL based on ROC curve analysis.

Sera from patients treated with ipilumumab were tested for sCTLA4 levels by ELISA, and levels compared between those with and without clinical benefit. Mean sCTLA4 levels for the patients with benefit from ipilimumab was 2,417 pg/mL with median levels of 918 pg/mL (range 0–8, 930 pg/mL). Mean sCTLA4 levels for the patients without benefit from ipilimumab was 208 pg/mL with median levels of 0 pg/mL (range 0–995 pg/mL). The average levels of sCTLA4 in serum from patients with clinical benefit were higher than from those without clinical benefit (*p* < 0.05) (Figure 1).

To determine sCTLA4 levels predictive of patients with benefit, we performed ROC curve analysis, which predicted sCTLA4 levels of ≤200 pg/mL as a cutoff for prediction of those without benefit. Based on this cutoff, the sensitivity of sCTLA4 levels for prediction of clinical benefit was 77.8%, specificity was 80%, positive predictive value of sCTLA4 levels was 87%, and negative predictive value was 67%. Two-tailed Mann–Whitney test confirmed that sCTLA4 levels >200 were predictive of response to ipilimumab (Figure 1).

### OS of sCTLA4 levels ≤200 vs. >200

**Figure 2:**
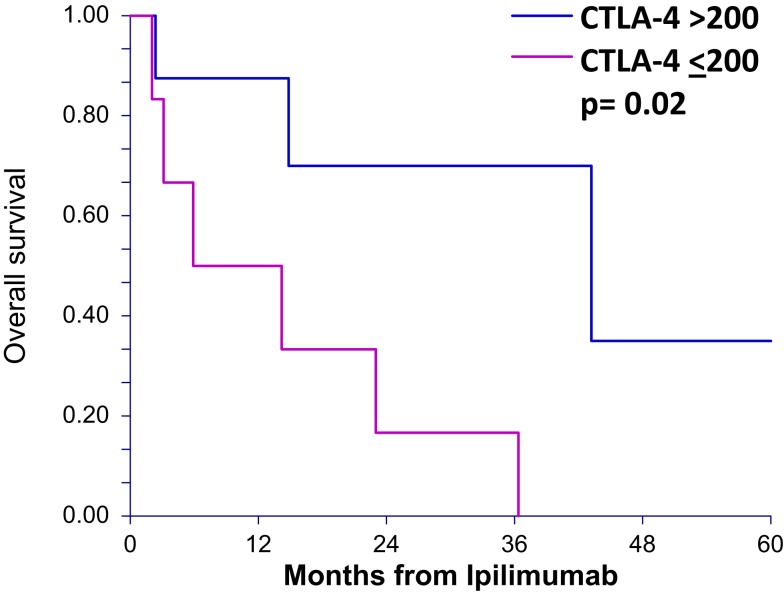
**Overall survival after ipilimumab treatment is greater in patients with serum CTLA4 levels above 200 pg/mL for ipilimumab-treated patients**. Overall survival curves (5 years) of patients treated with ipilimumab comparing those with greater than 200 pg/mL serum sCTLA4 to those with less than or equal to 200 pg/mL.

To determine whether patients treated with ipilimumab derive survival benefit based on sCTLA4 levels, we examined survival rates using a cutoff sCTLA4 level >200 pg/mL, determined by ROC analysis. Those with sCTLA4 levels >200 pg/mL had a higher percentage with 5-year OS (70 vs. 16.6% for sCTLA4 levels ≤200 pg/ml; *p* = 0.02). Median OS was 5.9 and 43.2 months for patients with sCTLA4 ≤200 pg/mL and sCTLA4 >200 pg/mL, respectively (Figure 2). Multivariable analysis showed that no covariate other than elevated sCTLA4 level was associated with prolonged 5-year OS (Table 3).

**Table 3:** **Univariable Cox Proportional hazard regression analysis of 5-year overall survival**.

Variable	*p*-Value	Hazard ratio	95% CI
Elisa <200 vs. >200	0.04	5.29	1.06–26.4
Male vs. female	0.97	0.97	0.26–3.66
Age at diagnosis	0.52	0.97	0.90–1.06
Breslow depth	0.67	1.02	0.94–1.11
Ulcerated vs. non-ulcerated	0.51^a^	NA	NA
M1abc	0.32		
M1a vs. M1c	0.20	3.19	0.55–18.59
M1b vs. M1c	0.64	0.60	0.07–5.35

*^a^By log-rank test (Hazard ratio approached zero so log-rank test was done instead)*.

*Multivariable analysis incorporating all the above variables showed that all the other variables except (ELISA) were not significant. Thus, the univariable model with ELISA is the final model*.

**Figure 3:**
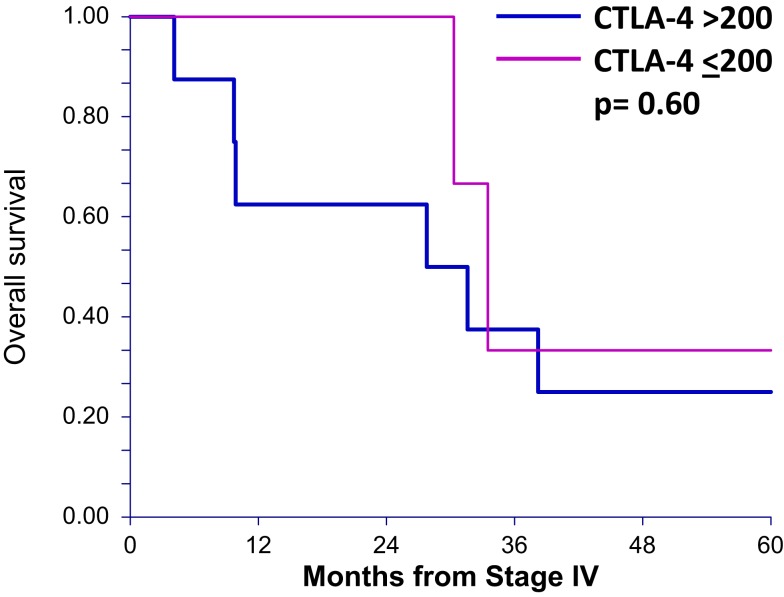
**Overall survival after stage IV diagnosis in patients not receiving ipilimumab treatment is not different in patients with serum sCTLA4 levels above 200 pg/mL**. Overall survival curves (up to 5 years) of patients who did not receive ipilumumab comparing those with greater than 200 pg/mL serum sCTLA4 to those with less than or equal to 200 pg/mL.

To determine whether sCTLA4 levels correlate with survival in general, rather than only in those patients who are treated with anti-CTLA4, we also tested stage IV melanoma patients who had not received ipilimumab (*n* = 11). Using the same cutoff of 200 pg/mL, the two survival curves were not statistically different, with 33.4 and 29.6 months median OS for patients with sCTLA4 ≤200 pg/mL and sCTLA4 >200 pg/mL, respectively (*p* = 0.60) (Figure 3).

## Discussion

Here, we report serum sCTLA4 levels of ipilimumab-treated patients with advanced melanoma from samples collected prior to ipilimumab therapy. Our preliminary findings show that elevated serum levels of sCTLA4 are associated with clinical benefit to ipilimumab in this initial cohort. Patients with elevated sCTLA4 also showed a significant survival benefit over those with low sCTLA4 levels (defined as <200 pg/mL). It is unclear, why some patients with melanoma have enhanced circulating levels of sCTLA4; however, increased levels of serum sCTLA4 have also been reported in patients with breast cancer (14). Elevated serum sCTLA4 may be due to increased secretion from T regulatory cells (Tregs), as these cells are a prominent source of sCTLA4 (15). Additional analysis of sCTLA4 levels from 11 non-ipilimumab-treated patients indicate levels of sCTLA4 alone are not correlated with survival. Taken together, these data suggest patients with higher circulating levels of sCTLA4 receive clinical benefit to a drug, which binds this target.

sCTLA4 has been shown to have an inhibitory effect on T cell responses. Neutralization of sCTLA4 using soluble isoform-specific antibodies increased T cell proliferation and cytokine production (12). In fact, sCTLA4 inhibited production of cytokines IFNγ, IL-2, IL-7, IL-13, and IL-17, yet activated the secretion of immunosuppressive cytokines TGFβ and IL-10 (12, 15). Aside from regulating cytokine production, there are a number of scenarios by which sCTLA4 might regulate the melanoma-specific T cell response. First, sCTLA4 might compete with CD28 and block T cell co-stimulation as does the membrane-bound form (11, 16). In addition, sCTLA4 can induce the enzyme indoleamine 2,3 dioxygenase (IDO), which catabolizes tryptophan, leading to an unfavorable microenvironment for effector T cells (17). Lastly, it has been shown that a recombinant form of sCTLA4 (CTLA4-Ig) can stimulate transport of the FoxO3 transcription factor to the nucleus (18). FoxO3 controls the magnitude of T cell responses by modulating the function of dendritic cells. Based on the ability of sCTLA4 to suppress T cell responses through mechanisms described above, the treatment of patients with ipilimumab may help to counteract sCTLA4-induced immune suppression and promote protective T cell responses.

As one of the first immune therapies to show survival advantage in randomized clinical trial (2), ipilimumab is an important advance in the treatment of metastatic melanoma. While ipilimumab demonstrates a significant improvement in OS for metastatic melanoma, ipilimumab is effective in less than 25% of patients with tumor shrinkage in only 6–11% of patients (19). In addition, ipilimumab-induced toxicities, usually autoimmune in nature, can occur in up to 60% of patients treated with significant morbidity. Ipilimumab toxicities range from mild side effects such as diarrhea, rash, and weakness to irreversible panhypopituitarism, colitis with perforation, and even death (20).

Ipilimumab has become a standard therapy for Stage IV melanoma worldwide. Currently it is being evaluated in multiple clinical trials and investigated for adjuvant therapy in high risk Stage III and resected metastatic patients in EORTC 8071 as well as ECOG E1609 (21). Clinical trials evaluating ipilimumab as combination therapy with other agents including programed death-1 (PD-1) pathway inhibitors are also currently underway (22). Ipilimumab is also being used in clinical trials in cancers other than melanoma such as lung (23) and prostate (24). As with any potentially toxic therapy with relatively low response rates, a predictive test to identify patients who may benefit is desired. Previous studies have examined tumor sample microarray analysis of mRNA expression of immune related genes (25), NY-ESO-1 antibody seropositivity (26), and absolute lymphocyte count (27); however, the ability to discriminate those who might benefit from ipilimumab is still out of reach. Our preliminary findings provide rationale for further prospective validation in melanoma patients as well as those with other malignancies for which ipilimumab is being tested.

## Authors Contribution

Acquisition of data: Anna M. Leung, Donald L. Morton, Junko Ozao-Choy, Mark B. Faries, Peter A. Sieling, Agnes Fermin Lee, Romela Irene Ramos. Analysis and interpretation of data: all authors. Drafting of manuscript: Anna M. Leung, Peter A. Sieling, Agnes Fermin Lee, Delphine J. Lee. Critical review of intellectual content: all authors. Statistics: Myung Shin-Sim. Funding: Anna M. Leung, Donald L. Morton, Mark B. Faries, Delphine J. Lee. Administrative and technical support: Anna M. Leung, Peter A. Sieling, Agnes Fermin Lee, Romela Irene Ramos, Delphine J. Lee. Study supervision: Donald L. Morton, Mark B. Faries, Delphine J. Lee.

## Conflict of Interest Statement

Dr. O’Day has the following disclosures with Bristol Meyer Squibb to report. He receives research funding support, participates in BMS Speaker’s Bureau, and serves as a consultant. The remaining authors have no disclosures.
